# ERβ and ApoE isoforms interact to regulate BDNF–5-HT_2A_ signaling and synaptic function in the female brain

**DOI:** 10.1186/s13195-017-0305-3

**Published:** 2017-09-21

**Authors:** Anindit Chhibber, Liqin Zhao

**Affiliations:** 1 Department of Pharmacology and Toxicology, School of Pharmacy, University of Kansas, 1251 Wescoe Hall Drive, Malott Hall Room 2046, Lawrence, KS 66045 USA; 2 Neuroscience Graduate Program, University of Kansas, Lawrence, KS USA

**Keywords:** Alzheimer’s disease (AD), Depression, Apolipoprotein E (ApoE), Estrogen receptor β (ERβ), Brain-derived neurotrophic factor (BDNF), 5-Hydroxytryptamine 2A (5-HT_2A_), Synaptic function

## Abstract

**Background:**

Depression has been reported to be commonly manifested in patients with Alzheimer’s disease (AD) and is considered a risk factor for AD. The human apolipoprotein E (ApoE) gene exists in three major isoforms (coded by ε2, ε3, and ε4), and the ε4 allele has been associated with a greater incidence of both depression and AD. Although mounting evidence points to the potentially complex interaction between these two brain disorders in which ApoE might play a role, the underlying mechanisms are largely unknown.

**Methods:**

Using human ApoE2, ApoE3, and ApoE4 gene-targeted replacement (hApoE-TR) mouse models, we investigated the role of ApoE isoforms and their potential interactions with estrogen receptor β (ERβ) signaling in modulating the brain mechanisms involved in depression.

**Results:**

Our initial analyses in 6-month-old female hApoE-TR mice demonstrated that ApoE influenced the expression of brain-derived neurotrophic factor (BDNF) and the 5-hydroxytryptamine 2A (5-HT_2A_) serotonin receptor in an isoform-dependent manner, with the ApoE4 brain exhibiting the lowest level of BDNF and the highest level of 5-HT_2A_. In addition, both presynaptic and postsynaptic proteins were downregulated, indicating a synaptic deficit in ApoE4 brains. Our subsequent analyses revealed that a 3-month chronic treatment with an ERβ-targeted (83-fold selectivity over ERα) phytoestrogenic diet induced several changes in ApoE2 and ApoE3 brains, including a significant decrease in the expression of 5-HT_2A_ receptors and an increase in BDNF/tropomyosin receptor kinase B and synaptic proteins. In contrast, ApoE4 brains were largely unresponsive to the treatment, with an increase only in select synaptic proteins in the treated group.

**Conclusions:**

Taken together, these results indicate that ApoE4 negatively impacts BDNF–5-HT_2A_ signaling in the female brain, which could in part underlie the ApoE4-mediated increased risk for depression. In a larger context, this mechanism could serve as a molecular link between depression and AD associated with ApoE4. Enhancing ERβ activity could provide a greater therapeutic benefit to non-ApoE4 carriers than to ApoE4 carriers in interventions for these brain disorders.

## Background

Depression manifests in up to 50% of patients with Alzheimer’s disease (AD) [1–3] and is associated with increased neurological impairment [4] and mortality [5]. In addition, depression is an independent risk factor for the development of mild cognitive impairment (MCI) and for the progression from MCI to AD [6], contributing to at least a twofold increased AD risk compared with control subjects without depression [7–10]. Furthermore, the severity of the depressive phenotype (measured by the number of depressive symptoms in patients with depression) has been shown to be directly associated with an increased risk of developing AD, with each depressive symptom increasing the risk of AD development by approximately 20% compared with control subjects without depression [11]. Although a number of clinical studies have indicated the interaction between depression and AD, the underlying pathophysiological mechanisms are not understood.

Human apolipoprotein E (ApoE) is a 299-amino-acid lipoprotein containing an N-terminal receptor-binding region (amino acids 136–150) and a C-terminal lipid-binding region (amino acids 244–272), which are linked by a flexible hinge region [12]. The human ApoE gene, located on chromosome 19, comprises four exons, which are transcribed into an 1180-nucleotide-long ApoE messenger RNA (mRNA) transcript. Single-nucleotide polymorphisms in exon 4 of the ApoE gene result in the formation of three major ApoE protein isoforms that differ by one or two amino acids at residues 112 and 158: ApoE2 (Cys112, Cys158), ApoE3 (Cys112, Arg158), and ApoE4 (Arg112, Arg158), which have population frequencies of 8%, 75%, and 14%, respectively [13]. These amino acid differences have been shown to significantly alter ApoE protein structure and function [14, 15]. For instance, in addition to the two structural domains (i.e., receptor- and lipid-binding domains) present in ApoE2 and ApoE3, ApoE4 contains an extra domain interaction between Arg61 and Glu225 that renders ApoE4 more susceptible to proteolysis [14, 15].

The three ApoE variants have been reported to differentially impact the development and progression of late-onset Alzheimer’s disease (LOAD). Possession of one ApoE4 allele increases the risk of LOAD development by two- to threefold, and possession of two ApoE4 alleles increases the risk for LOAD by 12-fold, compared with non-ApoE4 carriers [16]. In contrast, possession of the ApoE2 allele has been reported to reduce the risk of LOAD by 50% [13, 17]. In addition, one copy of the ApoE4 allele shifts the risk curve for AD development 5 years earlier, and two copies of the ApoE4 allele shifts the curve 10 years earlier, whereas one copy of the ApoE2 allele shifts the curve 5 years later [18, 19]. Although several genome-wide association studies have identified many other loci associated with the development of LOAD, all of the studies have confirmed that ApoE is the strongest genetic risk factor associated with the development of LOAD [20–22].

In addition to contributing significantly to AD risk, ApoE has been implicated in the etiology of depressive disorders. The mean age of onset of depressive episodes in ApoE4 carriers was found to be significantly lower than the mean age of onset in non-ApoE4 carriers [23]. Moreover, ApoE4 was reported to be highly prevalent in patients with depression compared with control subjects without depression [24] and is significantly associated with the incidence of minor depression, severe depression, and depressive symptoms of any kind [25]. These data suggest that the ApoE4 allele may be a potential biomarker useful for identifying people who are at high risk of developing clinical depression [25, 26]. Similar to its protective role in LOAD, the ApoE2 allele has been associated with reduced risk for the onset of depressive disorders [27, 28].

Collectively, clinical studies have pointed toward a possible complex interaction of ApoE, AD, and depression. The goal of this study was to elucidate the mechanisms that underlie the association between the ApoE genotype and depression by examining the three ApoE isoforms’ differential modulation of brain-derived neurotrophic factor (BDNF) signaling, serotonergic signaling, and synaptic function. Moreover, to extend our previous findings on the potential role of estrogen receptor β (ERβ) in depression [29], we also analyzed ERβ-mediated effects on these signaling pathways in the presence of ApoE isoforms. The data provide a probable mechanistic rationale for the differential risk of depression associated with ApoE genetic variants, in which ERβ might play a role.

## Methods

### Animal models

The use of animals was approved by the Institutional Animal Care and Use Committee at the University of Kansas and followed National Institutes of Health guidelines for the care and use of laboratory animals. The study was carried out using human ApoE2, ApoE3, and ApoE4 gene-targeted replacement (hApoE2-TR, hApoE3-TR, and hApoE4-TR) mouse models. These mouse lines were created by gene targeting and carry one of the three human ApoE alleles in place of the endogenous murine ApoE gene while retaining the endogenous regulatory sequences required for modulating hApoE expression. These mice share a C57BL/6 J genetic background and express the human ApoE protein at physiological levels; thus, they provide a complete in vivo system that allows direct measurement and comparison of hApoE isoform-specific effects [30, 31]. For hApoE isoform comparison studies, cortical tissues were collected from 6-month-old hApoE2-TR, hApoE3-TR, and hApoE4-TR female mice (*n* = 5 for each isoform group). Dietary treatment studies were conducted with 3-month-old hApoE2-TR, hApoE3-TR, and hApoE4-TR female mice for a period of 3 months (*n* = 5 for each isoform/treatment group).

### Animal treatment

Two rodent diets, a base/control diet and a phytoestrogenic estrogen receptor β-selective modulator (phyto-β-SERM)-supplemented diet, were custom-manufactured by Harlan Laboratories (Madison, WI, USA). Phyto-β-SERM is a rationally designed combination of three clinical phytoestrogens (genistein, daidzein, and equol) and exhibits an 83-fold binding selectivity for ERβ over ERα [32]. We have previously demonstrated that dietary supplementation with a clinically relevant dose of phyto-β-SERM resulted in significantly improved neurological outcomes in both menopausal and AD mouse models, without inducing estrogenic effects in reproductive tissues [33, 34]. The base/control diet was prepared from the Teklad Global 16% Protein Rodent Diet (Harlan Laboratories), which was ground and repelleted. This diet has a fixed formula and is nutritionally balanced, containing 16% protein and 3.6% fat. It supports the growth and maintenance of rodents and does not contain alfalfa or soybean meal, thus minimizing the levels of natural phytoestrogens. The phyto-β-SERM diet was prepared by adding equal parts of genistein, daidzein, and equol (LC Laboratories, Woburn, MA, USA) to the base diet; a total of 100 mg (genistein, daidzein, and equol) was added per 1000-g diet. This diet would deliver to each mouse a daily intake of 0.25 mg of added phyto-β-SERM formulation (genistein, daidzein, and equol) or 10 mg/kg (body weight [BW]) per mouse per day, assuming a 25-g mouse eating a 2.5-g diet per day. The diet was designed to deliver to the mice a total amount of added phytoestrogens that is biologically equivalent to a daily intake of 50 mg in humans. The conversion of a human dose to a mouse-equivalent dose was based on the conversion factor of equivalent surface area dose from human to mouse: 50 mg/60 kg (BW, human) × 12 (human-to-mouse conversion factor) = 10 mg/kg (BW, mouse). Three-month-old hApoE2-TR, hApoE3-TR, and hApoE4-TR female mice were fed one of the two custom diets for 3 months, and at the end of the treatment, mice were killed and their brain tissues were immediately collected.

### Protein extraction

For tissue protein extraction, 30 mg of cortical tissue samples were homogenized using a Bullet Blender 24 homogenizer (Next Advance, Troy, NY, USA) with Pierce T-PER Tissue Protein Extraction Reagent (Thermo Fisher Scientific, Rockford, IL, USA) supplemented with protease and phosphatase inhibitors (Roche Applied Science, Indianapolis, IN, USA) and 100 μl of 0.5-mm glass beads (Next Advance) at speed 8 for 3 minutes at 4 °C, followed by centrifugation at 12,000 rpm for 8 minutes at 4 °C. Supernatants were transferred to new microcentrifuge tubes, and protein concentrations were determined using a bicinchoninic acid assay (Thermo Fisher Scientific).

### Western blot analysis

Equal amounts of total protein (20 μg/lane) were loaded and separated by 10% glycine sodium dodecyl sulfate-PAGE. Resolved proteins were transferred to 0.2-μm pore-sized polyvinylidene difluoride membranes (Bio-Rad Laboratories, Hercules, CA, USA) and blocked with 5% blotting grade blocker (Bio-Rad Laboratories) in Tris-buffered saline (TBS) with Tween 20 (TBST; 100 ml of 10× TBS [200 mM Tris, 1.5 mM NaCl, pH 7.6], 10 ml of 10% Tween 20, 890 ml of double-distilled H_2_O) for 1 h at room temperature (RT), followed by incubation with customized dilutions of primary antibodies at 4 °C overnight. Following overnight incubation, membranes were washed three times for 10 minutes each in TBST at RT, followed by incubation with horseradish peroxidase-conjugated secondary antibody (1:5000; Thermo Fisher Scientific) for 1 h at RT. Blots were again washed three times for 10 minutes each in TBST. Bands were visualized using chemiluminescence with an enhanced chemiluminescence detection kit (Bio-Rad Laboratories) and scanned using the C-DiGit Blot Scanner (LI-COR Biosciences, Lincoln, NE, USA). Relative intensities of the immunoreactive bands were quantified using image-digitizing software (Image Studio version 4.0; LI-COR Biosciences). Membranes were stripped in 5 ml of Restore PLUS Western Blot Stripping Buffer (Thermo Fisher Scientific) for 8 minutes at RT and reprobed with the indicated loading control. The following primary antibodies were used: rabbit polyclonal anti-BDNF (1:500; Santa Cruz Biotechnology, Dallas, TX, USA), rabbit polyclonal anti-tropomyosin receptor kinase B (anti-TrkB, 1:1000; Abcam, Cambridge, MA, USA), mouse monoclonal anti-β tubulin (1:3000; Thermo Fisher Scientific, Waltham, MA, USA), rabbit polyclonal anti-PSD95 (1:500; Alomone Labs, Jerusalem, Israel), rabbit monoclonal anti-synaptophysin (1:1000; Abcam), mouse monoclonal anti-SHANK3 (1:1000; NeuroMab/Antibodies Inc., Davis, CA, USA), rabbit polyclonal anti-synaptobrevin 2 (1:1000; Enzo Life Sciences, Farmingdale, NY, USA), rabbit polyclonal anti-5-hydroxytryptamine (serotonin) (anti-5-HT_1A_, 1:1000; Abcam), and rabbit polyclonal anti-5-HT_2A_ (1:20,000; a generous gift from Dr. Nancy Muma).

### Statistical analysis

Data were presented as mean ± SD. For comparisons between two groups, Student’s *t* test was used; for comparisons involving multiple groups, one-way analysis of variance with Tukey’s or Dunnett’s post hoc test was used. All statistical analyses were performed with Prism version 5.0 software (GraphPad Software Inc., La Jolla, CA, USA). Statistical significance was indicated by *p < 0.05, **p < 0.01, and ***p < 0.001.

## Results

### BDNF but not TrkB receptor expression is affected by ApoE in an isoform-dependent manner

**Fig. 1:**
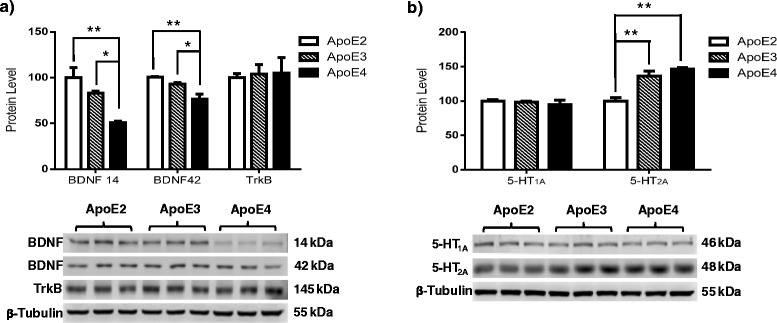
Brain-derived neurotrophic factor (BDNF) and 5-hydroxytryptamine 2A (5-HT_2A_) expression levels are modulated by apolipoprotein E (ApoE) in an isoform-dependent manner. Expression levels of (**a**) BDNF and tropomyosin receptor kinase B (TrkB) and (**b**) 5-HT_1A_ and 5-HT_2A_ were examined in the cortices of 6-month-old human ApoE2, ApoE3, and ApoE4 gene-targeted replacement female mice. The integrated density value of the bands in Western blots was determined using densitometry, and data were normalized to an internal loading control (β-tubulin) and to the ApoE2 group. Data are shown as mean ± SD (*n* = 5). *p < 0.05, **p < 0.01 by one-way analysis of variance with Tukey’s post hoc test

To elucidate the role of ApoE isoforms in regulating the BDNF-TrkB pathway, we harvested cortical tissues from 6-month-old hApoE-TR female mice and probed for BDNF and TrkB immunoreactivity. The data indicate a significant decrease in BDNF expression levels in ApoE4 animals compared with ApoE2 and ApoE3 animals, whereas no significant differences were found when we compared ApoE2 with ApoE3 animals (Fig. 1a) [BDNF 14: *F*(2,6) = 15.07, *p* = 0.0046; ApoE2 vs ApoE3, *p* = 0.2278; ApoE2 vs ApoE4, *p* = 0.0040; ApoE3 vs ApoE4, *p* = 0.0285; BDNF 42: *F*(2,6) = 13.25, *p* = 0.0063; ApoE2 vs ApoE3, *p* = 0.2929; ApoE2 vs ApoE4, *p* = 0.0007; ApoE3 vs ApoE4; *p* = 0.0025]. In addition, the data revealed no differential regulation of TrkB receptor expression levels among different ApoE isoforms (Fig. 1a) [TrkB, *F*(2,6) = 0.0478, *p* = 0.9535].

### 5-HT_2A_ but not 5-HT_1A_ receptor expression is affected by ApoE in an isoform-dependent manner

In addition to BDNF/TrkB expression, we also examined the probable regulation of serotonergic signaling by different ApoE isoforms. Because 5-HT_1A_ and 5-HT_2A_ are the best-characterized serotonergic receptors in the field of depression, we focused our examination on the expression levels of these two receptors [35, 36]. The data indicate no significant regulation of 5-HT_1A_ receptor expression by ApoE isoforms (Fig. 1b) [*F*(2,6) = 0.4805, *p* = 0.6404]. However, we observed differential regulation of 5-HT_2A_ receptor expression among ApoE isoforms. Specifically, 5-HT_2A_ expression increased by 15–20% in ApoE3 brains compared with ApoE2 brains, whereas ApoE4 brains expressed 30% more 5-HT_2A_ than ApoE2 brains (Fig. 1b) [*F*(2,6) = 20.03, *p* = 0.0022; ApoE2 vs ApoE3, *p* = 0.0084; ApoE2 vs ApoE4, *p* = 0.0022; ApoE3 vs ApoE4, *p* = 0.3951].

### Select presynaptic proteins are modulated by ApoE in an isoform-dependent manner

**Fig. 2:**
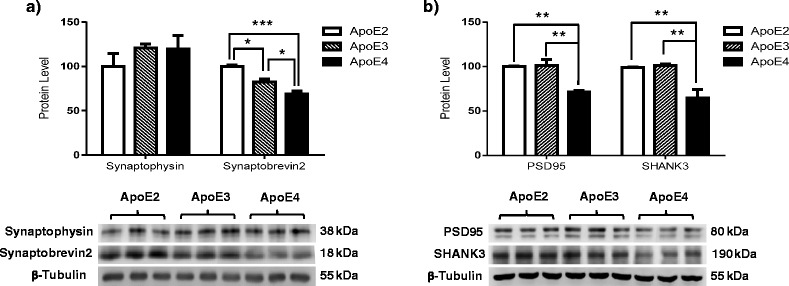
Pre- and postsynaptic proteins are differentially modulated by apolipoprotein E (ApoE) isoforms. Expression levels of (**a**) presynaptic proteins synaptophysin and synaptobrevin2, and (**b**) postsynaptic proteins PSD95 and SHANK3 were examined in the cortices of 6-month-old human ApoE2, ApoE3, and ApoE4 gene-targeted replacement female mice. The integrated density value of the bands in Western blots was determined using densitometry, and data were normalized to an internal loading control (β-tubulin) and to the ApoE2 group. Data are shown as mean ± SD (*n* = 5). *p < 0.05, **p < 0.01, ***p < 0.001 by one-way analysis of variance with Tukey’s post hoc test

Depression has been shown to downregulate the expression levels of presynaptic proteins, a phenomenon that is reversed in patients treated with antidepressants [37]. To elucidate the possible differential regulation of presynaptic proteins among ApoE isoforms, we probed cortical tissues of hApoE-TR mice for synaptophysin (a synaptic vesicle structural protein) and synaptobrevin2 (a SNARE protein involved in the docking of synaptic vesicles to the terminal membrane). The results indicated that synaptophysin was not regulated by ApoE isoform, because no significant difference in expression levels of synaptophysin was found among the three animal groups (Fig. 2a) [*F*(2,6) = 0.8986, *p* = 0.4557]. In contrast, our data indicate that synaptobrevin2 levels were differentially modulated by ApoE isoforms. Specifically, the data demonstrated a 15% decrease in synaptobrevin2 expression in ApoE3 animals compared with ApoE2 animals, a 30% decrease in ApoE4 animals compared with ApoE2 animals, and a 15% decrease in ApoE4 animals vs ApoE3 animals (Fig. 2a) [*F*(2,6) = 29.16, *p* = 0.0008; ApoE2 vs ApoE3, *p* = 0.0122; ApoE2 vs ApoE4, *p* = 0.0007; ApoE3 vs ApoE4, *p* = 0.0365].

### Postsynaptic proteins are downregulated in ApoE4 brains

In addition to presynaptic proteins, the functions and expression levels of postsynaptic proteins have also been shown to be decreased in depression [38]. Therefore, to elucidate the possible differential regulation of postsynaptic proteins by ApoE isoforms, we examined the protein expression of PSD95 (a postsynaptic density protein) and SHANK3 (a multidomain scaffold protein of the postsynaptic density) in cortical tissues from hApoE-TR mice. The data indicate that PSD95 was differentially regulated by ApoE isoforms. Specifically, we observed a 25% decrease in the expression levels of PSD95 in ApoE4 animals compared with both ApoE2 and ApoE3 animals, with no significant difference occurring between ApoE2 and ApoE3 animals (Fig. 2b) [*F*(2,6) = 18.17, *p* = 0.0028; ApoE2 vs ApoE3, *p* = 0.9810; ApoE2 vs ApoE4, *p* = 0.0052; ApoE3 vs ApoE4, *p* = 0.0044]. Similar to these findings, SHANK3 expression levels decreased by 25% in ApoE4 animals compared with both ApoE3 and ApoE2 animals, with no difference occurring between ApoE2 and ApoE3 animals (Fig. 2b) [*F*(2,6) = 14.54, *p* = 0.0050; ApoE2 vs ApoE3, *p* = 0.9330; ApoE2 vs ApoE4, *p* = 0.0099; ApoE3 vs ApoE4, *p* = 0.0069].

### Phyto-β-SERM treatment increases BDNF/TrkB signaling in ApoE2 and ApoE3 brains but not in ApoE4 brains

**Fig. 3:**
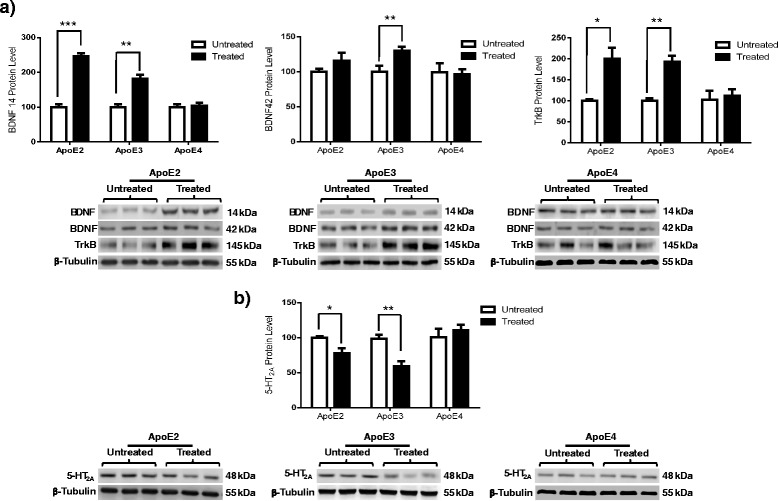
Estrogen receptor β activation modulates brain-derived neurotrophic factor (BDNF)/tropomyosin receptor kinase B (TrkB) and 5-hydroxytryptamine 2A (5-HT_2A_) pathways in apolipoprotein E2 isoform (ApoE2) and ApoE3 brains but not in ApoE4 brains. Three-month-old human ApoE2, ApoE3, and ApoE4 gene-targeted replacement female mice were treated with a phytoestrogenic estrogen receptor β-selective modulator (phyto-β-SERM)-supplemented diet or control diet for 3 months and killed at the age of 6 months. Cortical tissues were probed for (**a**) BDNF/TrkB and (**b**) 5-HT_2A_ immunoreactivity. The integrated density value of the bands in Western blots was determined using densitometry, and data were normalized to an internal loading control (β-tubulin) and to the untreated group of each genotype. Data are shown as mean ± SD (*n* = 5). *p < 0.05, **p < 0.01 by Student’s *t* test

Estrogen use has been implicated in alleviating the mood-related symptoms of depression as well as in improving the cognition- and memory-related deficits associated with AD. To examine the potential effects of ERβ-mediated signaling, hApoE mice were administered a control diet or an ERβ-targeted phyto-β-SERM-supplemented diet for 3 months and killed at 6 months of age. The data indicate that phyto-β-SERM treatment significantly increased the expression levels of BDNF in ApoE3 animals but not in ApoE2 or ApoE4 animals (Fig. 3a) (BDNF 14: ApoE2, *p* = 0.0001; ApoE3, *p* = 0.0006; ApoE4, *p* = 0.5652; BDNF 42: ApoE2, *p* = 0.2586; ApoE3, *p* = 0.0422; ApoE4, *p* = 0.7721). The data also revealed that the 3-month treatment with phyto-β-SERM increased the expression levels of TrkB receptors in ApoE2 and ApoE3 animals but not in ApoE4 animals (Fig. 3a) (ApoE2, *p* = 0.0283; ApoE3, *p* = 0.0033; ApoE4, *p* = 0.6400).

### Phyto-β-SERM treatment decreases 5-HT_2A_ expression in ApoE2 and ApoE3 brains but not in ApoE4 brains

In addition to the BDNF/TrkB pathway, we examined the effect of phyto-β-SERM treatment on the 5-HT_2A_ receptor expression levels. The data revealed decreased expression levels of 5-HT_2A_ receptor in ApoE2 and ApoE3 animals treated with phyto-B-SERM, but not in ApoE4 animals, compared with the control animals (Fig. 3b) (ApoE2, *p* = 0.0414; ApoE3, *p* = 0.0075; ApoE4, *p* = 0.3206).

### Phyto-β-SERM treatment alters expression of presynaptic proteins in an ApoE isoform-dependent manner

**Fig. 4:**
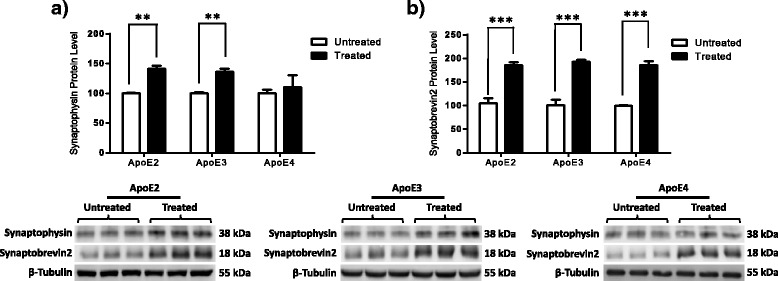
Estrogen receptor β activation leads to an upregulation of presynaptic proteins in an apolipoprotein E (ApoE) isoform-dependent manner. Three-month-old human ApoE2, ApoE3, and ApoE4 gene-targeted replacement female mice were treated with a phytoestrogenic estrogen receptor β-selective modulator (phyto-β-SERM)-supplemented diet or a control diet for 3 months and killed at the age of 6 months. Cortical tissues were probed for **a**) synaptophysin and **b**)﻿ synaptobrevin2 immunoreactivity. The integrated density value of the bands in Western blots was determined using densitometry, and data were normalized to an internal loading control (β-tubulin) and to the untreated group of each genotype. Data are shown as mean ± SD (*n* = 5). *p < 0.05, **p < 0.01, ***p < 0.001 by Student’s *t* test

In the context of the deleterious downregulation of functional presynaptic proteins in ApoE4 animals, we next examined whether ERβ agonism can modulate the aforementioned presynaptic proteins in 6-month-old female ApoE mice. Our data revealed that phyto-β-SERM treatment resulted in a significant increase in the expression levels of synaptophysin in ApoE2 and ApoE3 animals, but not in ApoE4 animals (Fig. 4a) (ApoE2, *p* = 0.0013; ApoE3, *p* = 0.0093; ApoE4, *p* = 0.6612). In contrast, the expression levels of S﻿ynaptobrevin2 increased in all animals treated with the phyto-β-SERM diet, regardless of ApoE isoform (Fig. 4b) (ApoE2, *p* = 0.0010; ApoE3, *p* = 0.0009; ApoE4, *p* = 0.0005).

### Phyto-β-SERM treatment alters expression of select postsynaptic proteins independent of ApoE status

**Fig. 5:**
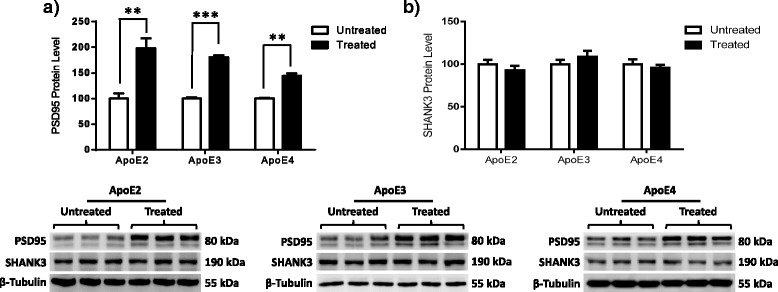
Estrogen receptor β activation leads to an upregulation of select postsynaptic proteins independent of apolipoprotein E (ApoE) status. Three-month-old human ApoE2, ApoE3, and ApoE4 gene-targeted replacement female mice were treated with a phytoestrogenic estrogen receptor β-selective modulator (phyto-β-SERM)-supplemented diet or a control diet for 3 months and killed at the age of 6 months. Cortical tissues were probed for **a**) PSD95 and **b**) SHANK3 immunoreactivity. The integrated density value of the bands in Western blots was determined using densitometry, and data were normalized to an internal loading control (β-tubulin) and to the untreated group of each genotype. Data are shown as mean ± SD (*n* = 5). *p < 0.05, **p < 0.01, ***p < 0.001 by Student’s *t* test

In addition to presynaptic proteins, our data revealed that ERβ agonism resulted in a significant increase in the expression levels of PSD95 in ApoE2, ApoE3, and ApoE4 animals (Fig. 5b) (ApoE2, *p* = 0.0076; ApoE3, *p* = 0.0001; ApoE4, *p* = 0.0013). In contrast, the expression levels of SHANK3 were not altered following chronic treatment with phyto-β-SERM, regardless of ApoE isoform (Fig. 5b) (ApoE2, *p* = 0.3485; ApoE3, *p* = 0.33; ApoE4, *p* = 0.5121).

## Discussion

Recent clinical studies have identified a significant association between depression and ApoE4, a major genetic risk factor for the development of AD [25]. Although the association has been well documented, the underlying molecular mechanisms leading to this probable association are unknown. On the basis of the literature and our own work, we hypothesize that the three ApoE isoforms differentially modulate neurotrophic and serotonergic pathways implicated in the pathophysiology of depression. The ApoE4 isoform causes significant dysregulation, thus increasing an individual’s risk of developing depression, and the ApoE2 isoform provides neuroprotection against the disease. To examine this hypothesis, we used 6-month-old human ApoE2, ApoE3, and ApoE4 gene-targeted replacement mouse models and found that BDNF and 5-HT_2A_ receptor expression was significantly impacted in an ApoE isoform-dependent manner. Specifically, BDNF significantly decreased, whereas 5-HT_2A_ receptor significantly increased, in ApoE4 animals compared with both ApoE2 and ApoE3 animals (Fig. 1). These data correspond with clinical findings of increased 5-HT_2A_ mRNA and protein expression levels [39–41] and decreased BDNF expression levels in patients with depression [42, 43]. Thus, the findings imply that the ApoE4-mediated increased risk of developing depression could be partially attributed to the upregulation of 5-HT_2A_ and downregulation of BDNF signaling in ApoE4 carriers compared with carriers of the other two isoforms.

The expression and function of synaptic proteins have also been found to be impaired in patients with depression [37]. To elucidate the probable regulation of synaptic proteins by ApoE status, we examined the modulation of the expression levels of both pre- and postsynaptic proteins in hApoE-TR animals (Fig. 2). The data indicate an isoform-based decrease in the expression levels of synaptobrevin2, a protein involved in vesicle docking and fusion, but not of synaptophysin, a protein involved in vesicular kinetics and recycling, when comparing ApoE4 animals with ApoE2 and ApoE3 animals, which suggests that ApoE isoforms regulate selective presynaptic proteins. In addition, the expression of the postsynaptic proteins PSD95 and SHANK3 was significantly reduced in ApoE4 animals compared with ApoE2 and ApoE3 animals. Collectively, these data indicate that synaptic function is compromised at both the pre- and postsynaptic levels in ApoE4 brains. The regulation of synaptic proteins in an ApoE isoform-dependent manner is well documented; ApoE4 has been associated with an overall decrease in synaptic protein content [44], including a substantial decrease in proteins such as synaptophysin [44], syntaxin [44], and PSD95 [45] in the presymptomatic stage of AD. Thus, it is highly probable that the deficit in synaptic strength and plasticity [46, 47], along with dysregulated BDNF–5-HT_2A_ signaling, in ApoE4 carriers increases their risk of developing depression, which, in the presence of environmental stressors, can lead to the manifestation of clinical depression.

Several lines of evidence indicate a probable interaction between estrogen signaling and ApoE isoforms in the regulation of neural activities in the central nervous system [48, 49]. For instance, 17β-estradiol increased the extent of neurite outgrowth in cultured adult mouse cortical neurons that expressed the human ApoE2 or ApoE3 genes, but it had no effect on neurons from nonexpressing mice or in those supplied with exogenous ApoE4 protein [50]. Similarly, in a familial AD mouse model expressing human ApoE gene isoforms, treatment with 17β-estradiol decreased amyloid deposition in the brains of ApoE2- and ApoE3-bearing mice, whereas amyloid deposition was increased in the brains of ApoE4-expressing mice [51]. Consistent with the findings in animal models, clinical studies demonstrated that estrogen therapy (ET) was associated with lesser cognitive decline in ApoE4-negative but not ApoE4-positive individuals [49, 52]. Together, these findings indicate that estrogen may have a dual effect in the brain modulated by ApoE genotype, as well as that it tends to exert a positive outcome when ApoE4 is absent, whereas an opposite outcome could happen when ApoE4 is present. This conclusion, however, is contradicted by studies in which researchers found that estrogen use was associated with a beneficial effect in ApoE4 carriers [53–55]. In addition to its protective role against AD, ET has been reported to exert positive effects in the treatment of depression and mood-related symptomatology [56–58]. Previous work [59–61], including our own [29], strongly indicates that ERβ signaling plays a key role in the mechanism of ET in depression. To further investigate the potential interaction between ERβ signaling and ApoE genetic status, we chronically treated hApoE-TR mice with an ERβ-targeted phyto-β-SERM diet [32–34] and analyzed changes in the expression levels of serotonergic, neurotrophic, and synaptic proteins. The results of these analyses provide strong evidence that ERβ agonism potently interacts with ApoE isoforms in modulating the brain mechanisms involved in depression (Figs. 3, 4 and 5).

## Conclusions

**Fig. 6:**
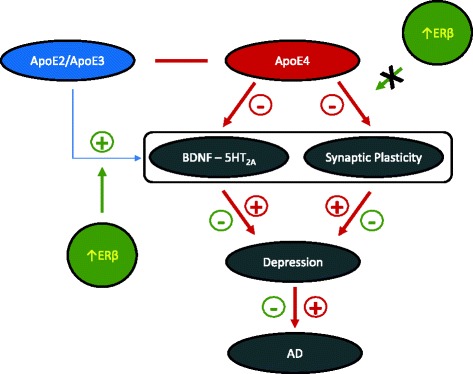
Estrogen receptor β (ERβ) interacts with apolipoprotein E (ApoE) in the regulation of brain-derived neurotrophic factor (BDNF)–5-hydroxytryptamine 2A (5-HT_2A_) signaling and synaptic function in the female brain. The findings presented indicate that ApoE isoforms differentially modulate the BDNF–5-HT_2A_ signaling and synaptic function in the female brain. Specifically, compared with ApoE2 and ApoE3, the presence of the ApoE4 allele exerts a largely negative impact on these signaling pathways, which predisposes the brain to an increased risk for depression, which further increases the risk for Alzheimer’s disease (AD). ERβ signaling positively modulates these brain pathways primarily in ApoE2 and ApoE3 brains, whereas it has a minimal role in the ApoE4 brain, implicating the potential interaction of ERβ signaling and ApoE isoforms in the modulation of certain functions in the female brain

Our findings illustrate a possible mechanism involving BDNF–5-HT_2A_ signaling pathways by which ApoE isoforms confer differential risk for depression (Fig. 6). In a larger context, this mechanism could serve as a link between depression and AD associated with ApoE4. In addition, our findings suggest that enhancing ERβ activity could provide a greater therapeutic benefit for ApoE2 and ApoE3 carriers than for ApoE4 carriers in interventions for depression. These preliminary data warrant further in-depth investigations of the pharmacological and behavioral relevance of the molecular differences identified in this study in depression models.

## Abbreviations

AD: Alzheimer’s disease
ApoE: Apolipoprotein E
BDNF: Brain-derived neurotrophic factor
BW: Body weight
ER: Estrogen receptor
ET: Estrogen therapy
5-HT: 5-Hydroxytryptamine (serotonin)
LOAD: Late-onset Alzheimer’s disease
MCI: Mild cognitive impairment
mRNA: Messenger RNA
Phyto-β-SERM: Phytoestrogenic estrogen receptor β-selective modulator
RT: Room temperature
TBS: Tris-buffered saline
TBST: Tris-buffered saline with Tween 20
TR: Gene-targeted replacement
TrkB: Tropomyosin receptor kinase B
